# Hydrophobicity Strategies of Starch-Based Films: Recent Advances and Perspectives

**DOI:** 10.3390/polym18040490

**Published:** 2026-02-15

**Authors:** Elsa F. Vieira, Tomás Amaral, Valentina F. Domingues, Cristina Delerue-Matos

**Affiliations:** REQUIMTE/LAQV, Instituto Superior de Engenharia do Porto, Instituto Politécnico do Porto Rua Dr. António Bernardino de Almeida 431, 4249-015 Porto, Portugal; tomasamaral2016@gmail.com (T.A.); vfd@isep.ipp.pt (V.F.D.);

**Keywords:** thermoplastic starch, starch-based films, hydrophobicity, chemical modification of starch, hydrophobic additives, nanofillers reinforcement, water vapor permeability, water contact angle

## Abstract

The rapid accumulation of plastic waste and the depletion of fossil resources have intensified global efforts to develop biodegradable polymeric materials derived from renewable feedstocks. In this context, starch-based films have emerged as one of the most promising alternatives to conventional petroleum-based plastics, owing to their wide availability, low cost, biodegradability, and ability to form continuous films using simple and scalable processing techniques. Starch is a naturally occurring polysaccharide composed primarily of amylose and amylopectin, whose molecular structure is rich in hydroxyl (–OH) groups. These functional groups promote extensive intermolecular hydrogen bonding, enabling starch gelatinization and film formation in aqueous systems. However, the same hydroxyl-rich structure confers a pronounced hydrophilic character, resulting in high moisture sensitivity, poor water vapor barrier properties, and limited dimensional stability under humid. Consequently, improving the hydrophobicity of starch-based films remains one of the most critical challenges for their practical application in food packaging. This review aims to summarize and critically discuss the main strategies reported for improving the hydrophobicity of starch-based films. The review focuses on composition and processing approaches, including (i) chemical modification of starch, (ii) incorporation of hydrophobic additives, (iii) reinforcement with natural fibers and nanocellulosic materials, (iv) polymer blending and multilayer/gradient architectures, and (v) processing strategies, including film homogenization, shear treatment and aging conditions. Emphasis is placed on the mechanisms governing hydrophobicity enhancement, comparative performance indicators, and current limitations.

## 1. Introduction

Plastics are indispensable in modern manufacturing due to their low density, chemical stability, mechanical durability, and cost-efficient large-scale production. Global plastic production has surpassed 390 million tonnes annually, with packaging constituting the dominant application sector [1,2]. However, the extreme environmental persistence of conventional petroleum-based plastics has raised serious ecological and societal concerns, as these materials resist biological degradation and accumulate across natural systems [3,4]. Plastic debris is now ubiquitous in terrestrial and aquatic environments, including agricultural soils, freshwater systems, marine ecosystems, and even remote polar regions, underscoring the global scale of the problem [5,6]. Modelling studies further predict that global plastic production may reach 900–1100 million tonnes per year by 2050 [2]. In this context, biodegradable polymers have emerged as one of the fastest-growing segments of the plastics market, with global production capacity exceeding 1.2 million tonnes in 2020 and continuing to expand [7].

Biodegradable polymers are generally classified as natural or synthetic biopolymers according to their origin and production pathway [8]. Natural biopolymers are derived from renewable biological resources, whereas synthetic biodegradable polymers are chemically synthesized from bio-based or fossil-derived monomers but remain susceptible to biological degradation. Representative examples include polylactic acid (PLA), polycaprolactone (PCL), polybutylene succinate (PBS), polybutylene adipate terephthalate (PBAT), and related aliphatic–aromatic copolyesters [9]. Polyhydroxyalkanoates (PHAs), produced by bacterial fermentation, constitute a distinct class of biodegradable polymers that combine renewable origin with thermoplastic processability [10].

Among natural biopolymers, starch-based materials have received sustained attention owing to their abundance, renewability, biodegradability, low cost, and wide availability from agricultural feedstocks [9,11,12,13]. A diverse range of starch-containing products is already commercially available, including compostable packaging, disposable items, and biomedical applications, most of which are produced as blends with synthetic polymers [14]. Industrial starch is primarily extracted from maize, rice, wheat, potato, and cassava [15,16].

**Figure 1:**
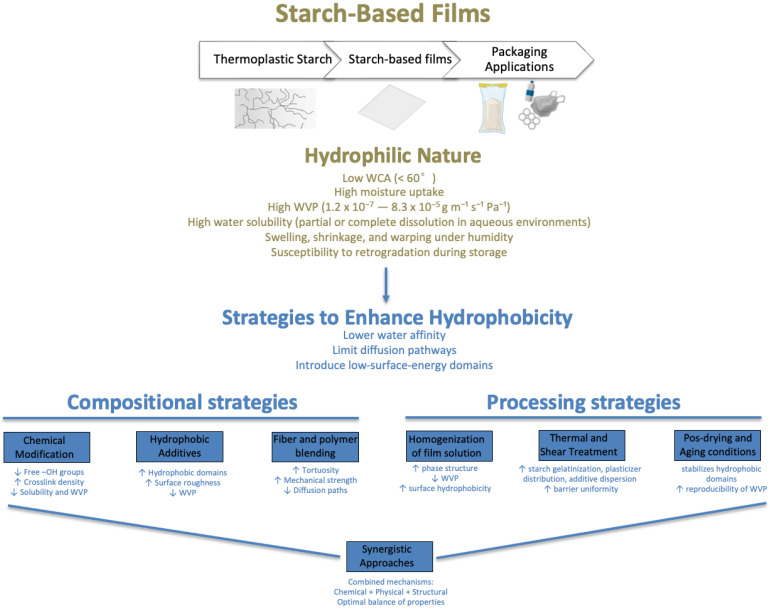
Hydrophilic character of starch-based films and approaches to increase hydrophobicity. Legend: water contact angle (WCA); water vapor permeability (WVP).

In its native form, starch exhibits extensive hydrogen-bond-induced crystallinity that precludes thermoplastic behavior. However, when processed with plasticizers under heat and shear, starch can be converted into thermoplastic starch (TPS) using conventional polymer-processing techniques such as extrusion, injection molding, and film casting [17]. During gelatinization, starch granules swell and disintegrate, amylose chains leach into the continuous phase, and amylopectin crystallites melt, forming a continuous polymeric network [15]. Despite these advantages, the intrinsic hydrophilicity of TPS severely limits its application in moisture-sensitive environments, particularly in food packaging. The high density of hydroxyl (–OH) groups along amylose and amylopectin chains promotes strong interactions with water molecules, resulting in high moisture uptake, elevated water vapor permeability, dimensional instability, and rapid deterioration of mechanical properties under humid [18,19], as illustrated in Figure 1. TPS also typically exhibits low thermal resistance, brittle mechanical behavior, and pronounced susceptibility to retrogradation during storage, further restricting shelf life and functional reliability [20].

To address these limitations, extensive research over the past decade has focused on enhancing the hydrophobicity and barrier performance of starch-based films. Proposed strategies include chemical modification of starch, such as esterification and crosslinking [13,14,15,16,17,18,19,20,21,22,23]; incorporation of hydrophobic additives including waxes [24], oils, and nanoparticles [18,25,26,27,28,29,30]; polymer blending [31,32], multilayer structures [33,34,35]; and reinforcement with natural fibers [18,36,37,38,39] or nanocellulosic materials [20,40,41]. These approaches aim to reduce the availability of hydrophilic sites, lower surface energy, and engineer microstructures that hinder water diffusion through the film matrix (Figure 1). Beyond formulation strategies, processing parameters, namely homogenization methods, drying regimes, film-forming techniques, and rheological behavior of film-forming solutions, play a decisive role in determining the final hydrophobic and barrier properties of starch-based films [11,12,13,42,43,44,45,46].

Parallel research on other natural polymers, including cellulose derivatives, chitosan, alginates, gelatin, zein, agar, and konjac glucomannan, has demonstrated promising barrier, antimicrobial, and active-packaging functionalities [19,30,32,34,42]. Nevertheless, starch remains particularly attractive as a platform material due to its low cost, global availability, compatibility with agricultural and food systems, and chemical versatility. The abundance of free hydroxyl groups in the starch chain enables facile functional-group substitution as well as oxidation to carbonyl and carboxyl derivatives [14].

The objective of this review is to critically examine recent advances in compositional design and processing strategies aimed at improving the hydrophobicity of starch-based films (Figure 1). The review synthesizes underlying mechanisms, quantitative performance trends, and comparative advantages of different approaches, while identifying current limitations and research gaps. A systematic literature search was conducted using Scopus and Web of Science, considering peer-reviewed articles published in English between 2015 and 2025. The remainder of this review addresses the structure and limitations of starch-based films, hydrophobicity-enhancement strategies, the role of fibrous and nanostructured reinforcements, synergistic hybrid approaches, applications in food packaging, and future perspectives.

## 2. Starch-Based Films: Processing and Properties

### 2.1. Starch Structure and Properties

Native starch is one of the most abundant renewable biopolymers and is obtained from a wide range of inexpensive agricultural resources, including maize, rice, wheat, potato, and cassava. Its botanical origin strongly influences granule size, crystalline organization, and the resulting performance of TPS materials [14,29,47]. These intrinsic structural differences translate into marked variation in gelatinization behavior, mechanical performance, and moisture sensitivity in starch-based films.

**Figure 2:**
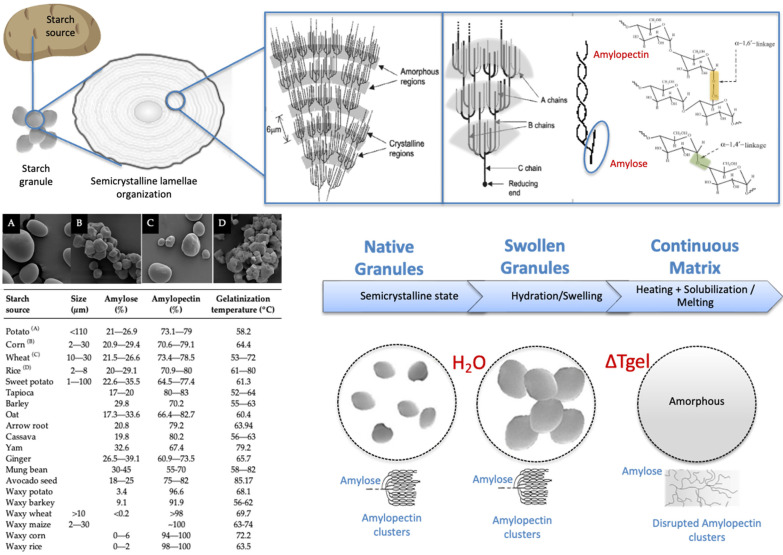
Structure and SEM images [53] of starch and its processing on TPS.

As illustrated in Figure 2, starch granules are primarily composed of amylose, a mostly linear α-(1→4)-linked glucan, and amylopectin, a highly branched macromolecule consisting of α-(1→4)-linked chains interconnected by α-(1→6) branch points [9,26]. Native starch displays a hierarchical semicrystalline organization with alternating crystalline and amorphous lamellae. The crystalline domains are mainly formed by ordered amylopectin double helices, whereas the amorphous regions are enriched in amylose and amylopectin branch points [26,48]. Among structural parameters, the amylose-to-amylopectin ratio is particularly critical, as it governs water uptake, crystallization kinetics, mechanical behavior, and barrier performance in starch-based films [17,20].

Beyond amylose content alone, botanical origin determines amylopectin chain-length distribution, crystalline polymorph type (A-, B-, or C-type), granule morphology, and the presence of endogenous lipids or phosphate groups, all of which contribute to structure–property relationships in TPS films. Cereal starches such as maize, wheat, and rice typically exhibit A-type crystallinity, characterized by densely packed double helices and lower hydration capacity, whereas tuber and root starches (e.g., potato and cassava) predominantly display B-type or mixed C-type crystallinity, with more open lattice structures and higher water accessibility. Consequently, potato and cassava starches generally show higher swelling power and water uptake during gelatinization, which facilitates film formation but also leads to increased moisture sensitivity and higher water vapor permeability in the resulting films [48]. Granule size further modulates processing and film performance. Potato starch, with large granules (20–100 µm), tends to gelatinize more heterogeneously and form films with higher thickness variability and greater susceptibility to microstructural defects, whereas rice and wheat starches, with smaller granules (<10 µm), often yield more homogeneous matrices and smoother film surfaces under comparable processing conditions [14,23]. These morphological differences directly influence diffusion pathways for water vapor and gases, thereby affecting barrier performance.

Differences in amylose content affect lamellar packing and the extent of residual crystallinity after processing, modulating diffusion pathways for water vapor and gases in TPS films [47]. In most cereal starches, amylopectin accounts for approximately 70–82% of total starch, while amylose typically represents 18–30% [14]. High-amylose starches (>50% amylose) and waxy starches (<1% amylose) represent useful compositional extremes for tailoring film properties [17]. High-amylose systems tend to form denser polymer networks with increased intermolecular entanglement, resulting in higher tensile strength and improved oxygen-barrier performance, albeit at the expense of flexibility and processability [49,50]. In contrast, waxy starches yield more flexible and transparent films due to their predominantly amorphous character but are more prone to rapid retrogradation and long-term instability, particularly under fluctuating humidity conditions [51].

Minor non-carbohydrate constituents inherent to specific botanical sources—such as lipids in cereal starches and phosphate monoesters in potato starch—can exert disproportionate effects on film performance. Endogenous lipids may form amylose–lipid inclusion complexes that restrict chain mobility and reduce swelling, leading to lower water uptake and, in some cases, reduced water vapor permeability (WVP). Conversely, phosphate groups in potato starch increase electrostatic repulsion and hydration capacity, enhancing swelling and film extensibility but generally increasing moisture sensitivity and permeability [52]. These compositional nuances help explain why starch films derived from different botanical origins can exhibit substantially different hydrophobicity, mechanical behavior, and aging responses even under identical formulation and processing conditions.

Upon heating in the presence of water, starch undergoes gelatinization: the hydrogen bonds stabilizing crystalline regions are disrupted, granules swell, amylose leaches into the continuous phase, and amylopectin crystallites melt (Figure 2). Depending on botanical origin and moisture content, this largely irreversible transition typically occurs between 65 and 90 °C [17,48,54]. As granules absorb water and swell, much of their original crystalline structure is lost. When shear is applied, swollen granules may be disrupted to varying degrees, depending on starch type and processing conditions [55]. During subsequent cooling and storage, dispersed starch chains gradually reassociate and reorganize into an ordered structure distinct from native starch, accompanied by a loss of water-holding capacity; this process is known as retrogradation [15]. Retrogradation adversely affects solubility, permeability, stability, and the mechanical properties of starch-based films [55,56].

TPS films consist of amorphous regions interspersed with residual or re-formed crystalline domains and are produced by converting native starch granules into a continuous polymeric matrix through the combined action of plasticizers (most commonly water, glycerol, and/or sorbitol), heat, and shear. This transformation disrupts the native semicrystalline granular structure, enabling melt or solution processability comparable to that of conventional thermoplastics [11,15]. However, the reduction in native crystallinity also increases the availability of hydroxyl groups, thereby intensifying hydrophilicity and moisture sensitivity. Plasticizers therefore play a decisive role in TPS structure and performance. Glycerol is widely used because it effectively reduces intermolecular hydrogen bonding and imparts flexibility, but its strong hygroscopicity and tendency to migrate during storage can compromise long-term performance. Alternative polyols such as sorbitol generally exhibit lower migration rates and can retard retrogradation, albeit at the cost of increased stiffness and melt viscosity, which may complicate processing [15,57]. Accordingly, recent research increasingly emphasizes co-plasticization strategies aimed at balancing flexibility, moisture resistance, and structural stability over time.

Despite its favorable processability and renewability, TPS remains intrinsically disadvantaged relative to conventional thermoplastics due to its high hydrophilicity, relatively high glass-transition temperature, brittle behavior under ambient conditions, and pronounced susceptibility to retrogradation. Moreover, retrogradation increases stiffness, reduces transparency, and degrades both mechanical and barrier properties [47,58]. These inherent limitations underscore the need for targeted strategies to enhance the hydrophobicity and long-term performance of starch-based films.

### 2.2. Production of Starch-Based Films

Starch-based films are produced using processing routes conceptually like those applied to petroleum-derived polymers, including solution casting, extrusion, thermocompression, and injection molding [14,20,38]. Some hydrophobicity effects of these methods are briefly summarized in Table 1. Alternative processing methods, such as injection molding, compression molding, foaming, and melt, wet, and electrospinning, have also been applied to produce starch-based films [18]. Nonetheless, starch processing is inherently more complex because native starch is not thermoplastic and exhibits high melt viscosity, extensive hydrogen bonding, and strong sensitivity to moisture and thermal properties. Hence, achieving reproducible film properties requires careful coordination of formulation parameters with processing conditions.

#### 2.2.1. Wet Processing

Solution casting is the most widely used method at laboratory scale due to its simplicity and suitability for systematic formulation screening. In this approach, starch is dispersed in excess water and heated (typically 70–95 °C) to induce gelatinization [14,17]. During heating, starch granules swell, crystalline lamellae are disrupted, amylose leaches into the continuous phase, and a viscous film-forming solution is obtained [55,59]. Despite these advantages, solution casting presents significant limitations for industrial translation. High water content results in long drying times, high energy consumption, and drying-induced heterogeneities, including thickness gradients, pore formation, and plasticizer migration. These effects strongly influence surface hydrophobicity and water vapor permeability (WVP), complicating direct comparison of formulation strategies unless drying and conditioning protocols are rigorously controlled [20,55,59].

#### 2.2.2. Dry Processing

The industrial production of starch-based films relies predominantly on dry thermoplastic routes, including extrusion followed by sheet or film extrusion, film blowing, thermocompression, or injection molding [14,17]. In these processes, starch is converted into TPS through the combined action of plasticizers, elevated temperature (typically 90–180 °C), and shear, which reduce intermolecular hydrogen bonding and enable flow below the degradation temperature [60,61]. Shear plays a critical role by accelerating granule restructuring and promoting homogeneous dispersion of hydrophobic additives and fillers [55]. However, excessive thermo-mechanical input may induce chain scission, plasticizer loss, or localized overheating, resulting in inferior mechanical and barrier properties [60,61]. Recent studies emphasize that extrusion parameters, including screw configuration, moisture content, and temperature profile, must be optimized in tandem with formulation to ensure consistent hydrophobic performance [44,62].

In the last decade increasing attention has been devoted to reactive extrusion, in which TPS formation is combined with chemical modification or crosslinking in a single processing step. This approach reduces solvent use and enables precise control over network formation, chain mobility, and dimensional stability [63,64]. Reactive extrusion has been successfully applied to introduce ester crosslinks or exploiting functionalities that reduce water solubility and improve moisture resistance [9,62,63]. From a hydrophobicity perspective, reactive extrusion is particularly attractive because it allows simultaneous dispersion of hydrophobic phases and fixation of the polymer network before phase separation or migration can occur. Nevertheless, reaction efficiency remains highly sensitive to moisture level and residence time, underscoring the importance of process–chemistry co-optimization [38,62,63,64].

#### 2.2.3. Role of Ingredients During Processing

Plasticizers are essential for TPS processing but are also a primary source of moisture sensitivity. An effective plasticizer is typically a low-molecular-weight compound that is compatible with the polymer matrix and increases intermolecular spacing [15]. In starch-based systems, polyhydroxy compounds, most notably glycerol and sorbitol, are widely used. Glycerol provides high flexibility and good processability but is strongly hygroscopic and prone to migration during storage [14]. In contrast, sorbitol and related polyols generally exhibit lower migration rates and can retard starch retrogradation, although they increase melt viscosity and stiffness, potentially complicating extrusion and filler dispersion [9,57]. Other plasticizers, such as citric acid, urea, and diacetin, have also been explored. Water can act as a plasticizer, but its volatility during high-temperature processing and storage compromises dimensional stability and may induce shrinkage [14,15].

To balance flexibility, barrier performance, and long-term stability, co-plasticization strategies combining water, glycerol, and sorbitol or alternative polyols are increasingly employed [26,60,65]. For instance, Abera et al. (2020) [66] reported that anchote starch films plasticized at 30–40 wt.% with glycerol, sorbitol, triethylene glycol, or ionic liquids were transparent, with 1-ethyl-3-methylimidazolium acetate yielding the highest flexibility. Glycerol- and triethylene glycol-plasticized films showed higher moisture uptake, whereas sorbitol produced non-sticky films over a wider concentration range. Ionic liquids, such as 1-ethyl-3-methylimidazolium acetate and 1-butyl-3-methylimidazolium chloride, have emerged as environmentally friendly alternatives, offering effective plasticization and, in some cases, superior flexibility compared with conventional polyols [67,68,69].

Films based solely on starch and plasticizers typically exhibit limited physicochemical and mechanical performance and are highly susceptible to retrogradation. Consequently, blending starch with co-biopolymers or incorporating fillers has been widely adopted to tailor composite film properties [47,68,69,70,71]. Such blending can modify gelatinization temperature as well as thermal and retrogradation behavior, leading to improved film performance [15]. In hydrophobicity-oriented formulations, emulsifiers, compatibilizers, or masterbatch approaches are often required to ensure uniform dispersion of lipid-based additives or nanofillers; inadequate dispersion during casting or extrusion can introduce microstructural defects that negate intrinsic hydrophobic effects and increase permeability [72].

#### 2.2.4. Post-Processing Conditioning

Regardless of processing route, TPS films are susceptible to retrogradation, a time-dependent reassociation and recrystallization of amylose and amylopectin chains during storage. Retrogradation alters stiffness, opacity, and barrier properties and is strongly influenced by residual moisture content, plasticizer distribution, and thermal history [55,72]. Consequently, post-processing conditioning under controlled temperature and relative humidity should be regarded as an integral stage of starch-based films manufacture, particularly when evaluating hydrophobicity strategies whose performance may evolve over time [64,73].

### 2.3. Physicochemical and Functional Properties of Starch-Based Films

#### 2.3.1. Thickness and Structural Uniformity

Film thickness is a key structural parameter influencing mechanical performance, barrier behavior, optical properties, and surface wettability of starch-based films [11,47,72]. In diffusion-controlled processes, permeability is inversely related to thickness when the microstructure is homogeneous; thus, uncontrolled thickness variations can confound structure–property relationships [57]. Thickness depends on solids content, processing geometry, drying conditions, and formulation variables such as plasticizer and filler loading. The incorporation of nanofillers (e.g., CNCs, CNFs, layered silicates) typically increases thickness by 15–25% at ~5–6 wt.% due to higher solids content and matrix densification driven by strong filler–starch interactions. Non-uniform drying, agglomeration, or phase separation may introduce local thickness gradients, leading to misleading barrier or wettability data. Accordingly, recent studies emphasize thickness normalization and correlation with microstructural analyses to ensure meaningful comparisons of hydrophobicity-enhancement strategies [57,72,73].

#### 2.3.2. Water Resistance and Surface Hydrophobicity

Water resistance of starch-based films is commonly assessed through water solubility, moisture uptake, WVP, and water contact angle (WCA). Native and plasticized starch films typically exhibit WVP values one to two orders of magnitude higher than conventional plastics, limiting their use in moisture-sensitive packaging [15].

Surface hydrophobicity is evaluated via WCA measurements: although WCA > 90° defines hydrophobicity, values above ~65–70° are often considered practically improved for starch-based films [15]. Chemical modification, incorporation of lipids or waxes, coatings, and nanoparticles can raise WCA to 80–120°, depending on formulation and surface morphology. However, durable water resistance requires stability under humidity cycling and handling, as plasticizer migration, surface rearrangement, and microcracking can rapidly degrade apparent hydrophobicity.

#### 2.3.3. Mechanical Properties

Mechanical performance, typically quantified by tensile strength (TS), Young’s modulus (YM), and elongation at break (EAB), is essential for handling and packaging integrity and is commonly measured following ASTM D882 [74]. Native starch films can exhibit high TS under dry conditions but are brittle and highly sensitive to humidity [14]. Increasing moisture content markedly reduces strength and stiffness. Plasticizers enhance flexibility and EAB but generally reduce TS and YM by weakening intermolecular interactions. Reinforcement with nanocellulose, inorganic nanoparticles, or natural fibers is an effective strategy to improve stiffness and strength and partially mitigate moisture-induced mechanical degradation. Nevertheless, excessive filler loading or poor dispersion can introduce stress concentrators and reduce EAB, compromising film integrity [15].

#### 2.3.4. Barrier Properties

Barrier performance against water vapor, oxygen, carbon dioxide, and aroma compounds is critical for food preservation. Gas and vapor transport in starch-based films follows a solution–diffusion mechanism governed by thickness, crystallinity, free volume, and environmental conditions. In the dry state, dense hydrogen-bonded networks can provide good oxygen barrier properties, but WVP remains high due to the strong affinity of starch for water [47]. Increasing humidity plasticizes the matrix, increases free volume, and sharply raises permeability [57,71]. While increased crystallinity can reduce gas permeability by creating tortuous diffusion paths, excessive crystallization or retrogradation may induce brittleness and microcracking, ultimately compromising barrier reliability [47,48]. Plasticizers generally increase WVP by enhancing hydrophilicity and free volume. Hybrid strategies combining chemical modification, hydrophobic additives, nanofillers, and multilayer structures are particularly effective in reducing WVP while retaining favorable oxygen barrier properties, enabling applications in dry and semi-moist food packaging.

#### 2.3.5. Optical Properties

Optical properties (transparency, opacity, haze, gloss, and UV–visible transmission) affect both consumer perception and protection against photo-induced degradation [9]. Transparency is typically quantified by UV–visible spectrophotometry, with opacity defined as absorbance at 600 nm normalized by thickness. Amylose-rich films tend to be more transparent due to their predominantly amorphous structure, whereas amylopectin-rich or highly crystalline systems scatter light more strongly [13,48]. Gelatinization generally improves transparency, while retrogradation reduces optical clarity during storage through chain reassociation. Plasticizers can enhance transparency at moderate levels but may cause phase separation or blooming at higher concentrations or under fluctuating humidity. Functional additives such as polyphenols, essential oils, pigments, and nanoparticles often reduce visible transparency but impart valuable UV-blocking, antioxidant, antimicrobial, or sensing functionalities relevant to active and intelligent packaging [19].

## 3. Hydrophilic Nature of Starch-Based Films

Table 2 summarizes the main limitations of native starch-based films and their molecular and structural origins. In unmodified systems, this hydrophilic nature leads to high moisture sorption, elevated WVP, and severe degradation of mechanical and barrier properties under humid conditions, thereby restricting their suitability for food packaging [15,57]. At the molecular level, hydrophilicity arises from the high density of hydroxyl (–OH) groups along amylose and amylopectin chains. While these groups form stabilizing hydrogen-bond networks in the dry state, exposure to moisture disrupts these interactions, causing swelling, plasticization, and increased chain mobility. As a result, stiffness and TS decrease, while the diffusion of water vapor and other permeants accelerates [13]. Microstructural heterogeneity further amplifies moisture sensitivity, as increases in relative humidity sharply raise free volume and diffusivity, leading to non-linear increases in WVP. Consequently, starch-based films may exhibit acceptable gas barrier performance under dry conditions but suffer rapid barrier deterioration at moderate to high humidity. In addition, time-dependent structural evolution imposes further constraints. Retrogradation, driven by chain reassociation and recrystallization during storage, progressively increases stiffness and brittleness while altering permeability and optical properties. Because this process is strongly coupled to moisture content, it can undermine long-term performance even when initial properties are adequate [11]. Collectively, these mechanisms highlight the need to enhance hydrophobicity and moisture resistance in starch-based films, motivating the targeted chemical, compositional, and structural modification strategies discussed in the following section.

## 4. Composition Strategies for Improving the Hydrophobicity of Starch-Based Films

Improving the hydrophobicity of starch-based films is a core objective for reducing moisture sensitivity while maintaining biodegradability and food-contact suitability. Composition-based approaches act mainly at molecular and microstructural levels to lower water affinity, limit diffusion pathways, or introduce low-surface-energy domains. The main strategies include (i) chemical modification, (ii) incorporation of hydrophobic additives, (iii) reinforcement with fibers and nanostructured fillers, and (iv) polymer blending, often combined to achieve synergistic gains in barrier and mechanical performance [47]. Table 3 links these approaches to the key limitations they address.

### 4.1. Chemical Modification of Starch

Chemical modification is one of the most direct and effective routes to reduce the intrinsic hydrophilicity of starch-based films. This approach relies on covalent alteration of amylose and amylopectin chains, in which hydroxyl groups are partially substituted or engaged in crosslinking reactions. By decreasing the number of free –OH groups available for hydrogen bonding with water, chemical modification typically lowers moisture uptake, water solubility, and WVP, while improving dimensional stability [13,21,47]. Table 4 summarizes typical ranges of hydrophobicity and barrier performance for chemically modified starch-based films reported in representative reviews. Because testing conditions and formulations vary (e.g., humidity, temperature, thickness, and methodology), the values should be interpreted as indicative trends rather than directly comparable benchmarks.

Among the various chemical pathways explored, esterification and crosslinking with polyfunctional organic acids have received sustained attention due to their effectiveness and compatibility with food-contact requirements. Citric acid is the most extensively studied crosslinker because it is bio-based, inexpensive, and of relatively low toxicity. Under appropriate thermal conditions, citric acid reacts with hydroxyl groups on adjacent starch chains to form ester linkages, producing a three-dimensional network that restricts chain mobility and limits water diffusion through the film matrix [77]. Starch films crosslinked with optimized levels of citric acid consistently exhibit reduced water solubility, lower WVP, increased water contact angle, and improved resistance to dimensional changes during humidity exposure. However, hydrophobicity enhancement is strongly dependent on crosslink density and reaction efficiency. Excess citric acid can result in incomplete esterification and residual carboxyl groups that act as secondary plasticizing sites, partially offsetting gains in moisture resistance and mechanical stability [77,78,79]. Consequently, precise control of formulation and processing conditions is essential.

Beyond citric acid, esterification with longer-chain hydrophobic moieties, such as fatty acid derivatives or alkyl anhydrides, has been shown to further reduce surface polarity and water affinity. These modifications often induce microphase separation and increased surface roughness, which together can yield water contact angles exceeding 100°, a range typically associated with highly hydrophobic [82,83]. While effective, such approaches may require reactive reagents or organic solvents, raising concerns related to cost, scalability, and regulatory acceptance for food packaging applications.

Alternative organic acids, including succinic, malic, and tartaric acids, have also been investigated as crosslinkers. Although generally less efficient than citric acid due to lower functionality, these acids can still improve moisture resistance and film integrity when combined with optimized plasticizer systems [85]. More recently, dual-modification strategies, such as combining mild esterification with phosphorylation, have been explored to balance hydrophobicity enhancement with mechanical flexibility and film cohesion [13,86]. Overall, chemical modification provides a powerful means of tuning starch hydrophilicity, but its success depends on achieving an appropriate balance between reduced water affinity, sufficient chain mobility, and retention of biodegradability and food-contact compatibility.

### 4.2. Incorporation of Hydrophobic Additives

Incorporation of hydrophobic additives is among the most scalable and industrially attractive strategies for improving moisture resistance in starch-based films. Unlike chemical modification, this approach preserves the native starch backbone and relies on physical interactions, phase organization, and microstructural effects to limit water transport [9,13,47]. Hydrophobic additives improve moisture resistance through three principal mechanisms: (i) formation of low-surface-energy domains that increase surface hydrophobicity; (ii) partial shielding of hydrophilic hydroxyl groups; and (iii) increased tortuosity of diffusion pathways for water vapor transport [13]. Effective performance depends critically on additive type, concentration, particle or droplet size, and dispersion quality. Table 5 presents a comparative overview of the impact of selected hydrophobic additives on the moisture resistance of starch-based films, based on findings reported in representative literature reviews. The reported ranges reflect variations in test conditions and formulation parameters and therefore should not be interpreted as directly comparable.

#### 4.2.1. Waxes, Lipids, and Oils

Barrier performance against water vapor, oxygen, carbon dioxide, and aroma compounds is critical for food preservation. Natural waxes and lipid-based additives are widely used due to their strong water-repellent character, biodegradability, and food-grade status. Commonly studied waxes include beeswax, candelilla wax and carnauba wax. When well dispersed, these materials form discontinuous hydrophobic domains that reduce surface energy and hinder water vapor diffusion, often yielding water contact angles above 90° and significant reductions in WVP [88,89]. Vegetable oils and essential oils also improve water resistance by shielding hydroxyl groups and disrupting hydrogen-bonded starch networks. At moderate loadings, oils can simultaneously reduce WVP and increase film flexibility through a secondary plasticizing effect [47,89]. Essential oils additionally impart antimicrobial and antioxidant functionality, making them attractive for active packaging applications [93]. However, excessive lipid content may cause phase separation, lipid migration, and long-term instability, underscoring the need for controlled formulation and processing [47,89].

#### 4.2.2. Organic Nanofillers

The use of nanoparticles (NPs) as polymer fillers is an established approach to enhance material properties at low filler loadings, with smaller, well-dispersed particles exerting stronger effects due to increased polymer–filler interfacial area [18]. NPs are commonly classified as inorganic (e.g., ZnO, TiO_2_, SiO_2_, MgO, CuO, ZrO2 NPs; nanoclays, such as montmorillonite, kaolinite; and halloysite nanotubes) or organic nanofillers (e.g., starch nanocrystals (SNC); starch nanoparticles (SNP); cellulose nanofibers (CNF); chitosan nanoparticles), or alternatively by their geometry and number of nanoscale dimensions. These nanomaterials provide a complementary route to starch-based films hydrophobicity enhancement by improving bulk barrier properties rather than surface chemistry alone.

Starch-based nanofillers has been shown to markedly improve mechanical strength, thermal stability, moisture resistance, oxygen barrier properties, and biodegradation behavior of starch-based films [94]. Starch nanocrystals (SNCs) are crystalline fragments formed by the removal of amorphous regions from starch granules and possess at least one nanoscale dimension (<100 nm). They can be derived from various botanical sources using several methods, most commonly sulfuric acid hydrolysis. However, this conventional approach is limited by aggregation, long processing times, low yields, and environmental concerns that hinder industrial implementation [18]. Thus, alternative production methods have been explored, including physical treatments (e.g., ultrasonication [95]; stirred media milling [96]; and high-pressure homogenization [97], and hybrid processes combining acid hydrolysis with mechanical or enzymatic treatments [98,99,100,101,102]. According to Oliveira et al. (2018) [103], the addition of 5% SNC to mango kernel starch film increased their TS and E in about 90% and 120% respectively and reduces their WVP in about 15%. On the other hand, the film EAB has been reduced to half the one for the unfilled film.

Chitosan nanoparticles, derived from chitin-modified chitosan, and synthesized using techniques such as tripolyphosphate crosslinking and emulsification, are effective bio-composite nanofillers due to their biodegradability, nontoxicity, and intrinsic antimicrobial activity. These fillers enhance gas and moisture barrier performance, thermal stability, mechanical strength, and antimicrobial efficacy of starch-based films, enabling shelf-life extension of perishable foods without synthetic preservatives [104,105]. For instance, Liu et al. (2023) [106] enhanced the hydrophobicity of corn starch films by incorporating oxidized debranched starch/chitosan nanoparticles. Films containing 3% nanoparticles exhibited a 3.6-fold increase in water contact angle (70° vs. 19.4°) compared with pure starch films, alongside markedly improved ultraviolet and light barrier properties.

#### 4.2.3. Inorganic Nanofillers

Inorganic nanoparticles (e.g., ZnO, TiO_2_, SiO_2_, MgO, CuO, ZrO_2_) are also widely investigated to enhance hydrophobicity of starch-based films [18]. When uniformly dispersed, these fillers significantly reduce WVP and can moderately increase WCA by increasing surface roughness and creating longer diffusion pathways. ZnO and TiO_2_ additionally provide antimicrobial and UV-protective functions, enhancing multifunctionality. However, nanoparticle aggregation can reduce TS and YM by forming rigid domains and decreasing interfacial contact [107]. Homogeneous dispersion is typically achieved through mechanical mixing techniques or ultrasonic sonication [18].

ZnO NPs have received more attention than other metal oxide nanomaterials. Anugrahwidya et al. (2022) [108] demonstrated that the incorporation of ZnO NPs enhanced the TS of starch films and extended bread shelf life. In a related study, ZnO NPs combined with fennel essential oil in cast potato starch films improved barrier performance by reducing water vapor and oxygen permeability while increasing TS [109]. The incorporation of TiO_2_ NPs into sago starch films increased TS and reduced EAB, water vapor, and oxygen permeability [110]. In another study, ZrO_2_ NPs combined with encapsulated essential oil improved the antioxidant, antibacterial, and mechanical properties of potato starch–apple peel films and extended quail meat shelf life by reducing moisture and WVP [111].

Carbon-based nanomaterials, including graphene oxide and carbon nanotubes, exhibit particularly strong barrier enhancement at low loadings due to their extremely high aspect ratio [18]. However, their effectiveness depends critically on surface functionalization and dispersion state, as oxygen-containing groups may introduce competing hydrophilic interactions. Agglomeration at higher nanoparticle contents leads to microstructural defects and diminished performance. Consequently, surface modification, compatibilizers, and optimized processing are essential to maximize barrier efficiency while preserving optical clarity and mechanical integrity [112].

Nanoclays (nanoscale lamellar silicates) represent a particularly effective class of nanofillers for addressing the limitations of starch-based matrices. Owing to their low cost, biocompatibility, and wide availability, nanoclays are extensively used in biodegradable films and coatings, as well as in synthetic polymer films, to enhance barrier performance and thermal stability [18]. Even low nanoclay loadings can reduce gas permeability in polymer films by factors ranging from 50 to 500 [113]. For instance, Ren et al. (2018) [114] showed that incorporating halloysite nanoclay into potato TPS films improved thermal stability and reduced moisture content irrespective of plasticizer type, while glycerol enabled better nanotube dispersion and greater TS enhancement than sorbitol due to stronger hydrogen bonding.

### 4.3. Reinforcement with Natural Fibers and Nanocellulosic Materials

Cellulose is the most abundant natural polysaccharide, consisting of linear β-(1→4)-linked D-glucose chains whose extensive hydrogen bonding imparts a highly crystalline structure. It is the primary structural component of plants, algae, and bacteria and can be sourced from trees, plant residues, and agricultural waste. In nature, cellulose assembles hierarchically from protofibrils into microfibrils and ultimately into fibers, with crystallinity stabilized by hydrogen bonding and van der Waals interactions, while amorphous regions arise from molecular disorder [18]. This polysaccharide has attracted significant attention in the form of cellulose nanomaterials, including cellulose nanocrystals (CNCs), cellulose nanofibrils (CNFs), and bacterial nanocellulose (BNC) to improves the functional performance of starch-based films primarily through microstructural densification and increased diffusion tortuosity rather than intrinsic chemical hydrophobicity. The ranges in Table 6 arise from differences in testing conditions and formulation parameters and are therefore not directly comparable.

CNFs typically have diameters of approximately 2–20 nm and lengths extending to several micrometers, with dimensions largely determined by their source, while CNFs contain both crystalline and amorphous regions and are commonly produced through high-shear mechanical treatments such as high-pressure homogenization, microfluidization, or grinding of cellulosic materials [116,117]. Overall, CNCs reduce WVP through their high crystallinity and rigid morphology, whereas CNFs form entangled networks that enhance TS and toughness [117].

Surface modification of fibers, through alkaline treatment or polyphenol functionalization, reduces surface polarity and improves fiber–matrix adhesion, leading to lower moisture uptake and improved durability [115]. However, excessive fiber loading may increase water affinity and induce aggregation, necessitating careful optimization. Potato starch films reinforced with pineapple leaf CNFs (3% *w*/*w*) exhibited enhanced UV resistance and improved water barrier properties [118]. Similarly, the addition of just 0.4 wt% henequen CNFs to TPS films increased TS and YM by 80% and 170%, respectively, while reducing water vapor and oxygen permeability by 86% and 94% [119].

### 4.4. Polymer Blending

Polymer blending represents another widely used composition strategy. Blending TPS with biodegradable polymers such as poly(lactic acid), poly(caprolactone), poly(vinyl alcohol), polyhydroxyalkanoates, or chitosan can substantially reduce moisture sensitivity and improve mechanical performance [15]. Chang et al. (2021) [120] reported that multilayer films based on glycerol-plasticized corn starch, maleated starch, and poly(butylene adipate-co-terephthalate) improved moisture and oxygen barrier properties by 86.8% and 74.3%, respectively, compared with neat starch films. Similarly, Trinh (2021) [33] showed that multilayer films incorporating maleated starch, nanoclay, corn starch, and poly(lactic acid) achieved moisture and oxygen barrier improvements of 1300% over native starch films and 3300% over poly(lactic acid) films, respectively. Hydrophobic phases impede water diffusion and may form surface-enriched layers during processing, further enhancing barrier properties [47,71]. However, immiscibility and weak interfacial adhesion often necessitate compatibilization strategies to achieve consistent performance.

Blends of chitosan with starches from various botanical sources have been extensively investigated. Overall, the combination of chitosan with starch—particularly starches with high amylose content—promotes the formation of strong intermolecular hydrogen bonds, leading to films with enhanced structural stability relative to their respective monolayer counterparts [121]. Incorporation of chitosan generally improves the mechanical performance and water barrier properties of starch-based films [122]. In corn starch–chitosan films, the addition of chitosan (degree of deacetylation 88%) results in increased TS and EAB, alongside reductions in YM and WVP compared to neat starch films [123]. Consistent trends are also observed in solubility behavior. Fonseca-García et al. (2021) [124] reported water solubility values of 21% for starch films and 76% for chitosan films (degree of deacetylation ≥75%), whereas blended films exhibited an intermediate solubility of 42%. A comparable intermediate behavior was likewise observed for water vapor permeability.

## 5. Processing Strategies for Improving the Hydrophobicity of Starch-Based Films

Variations in dispersion quality, phase morphology, and polymer-chain mobility introduced during processing can dominate moisture transport and mechanical response of starch-based films. Consequently, hydrophobicity enhancement must be understood not only as a compositional issue but also as a processing-controlled structure–property problem [47].

### 5.1. Homogenization of the Film-Forming System

Homogenization governs the spatial distribution of plasticizers, hydrophobic additives, and nanofillers within the starch matrix and is therefore a key determinant of microstructural uniformity. Insufficient homogenization leads to large droplets, filler agglomeration, and interfacial discontinuities that act as preferential pathways for moisture diffusion. In contrast, effective homogenization improves phase continuity and increases diffusion tortuosity, resulting in reduced WVP and enhanced surface hydrophobicity [9,124]. Several homogenization techniques have been optimized and evaluated for their effects on the hydrophobicity and barrier properties of starch-based films. Representative literature review is summarized in Table 7.

Rotor–stator mixing provides moderate shear and is suitable for simple formulations but often yields coarse dispersions in systems containing lipids or nanoparticles. High-pressure homogenization and ultrasonic treatment generate finer, more stable dispersions through intense shear and cavitation, promoting stronger interfacial interactions and more homogeneous polymer networks. However, excessive energy input may induce chain scission or reaggregation, underscoring the need for process optimization tailored to formulation complexity [115,125,126,127].

**Table 7:** Comparison of homogenization techniques used in the preparation of starch-based film-forming solutions and their effects on hydrophobicity and barrier properties.

Homogenization Technique	Shear/Energy Input	Main Functions	Effects on Film Microstructure and Hydrophobicity	Key Advantages	Main Limitations	Representative Literature Review
Rotor–Stator Homogenization	Low–moderate shear	Mixing, coarse dispersion, emulsification	Relatively large droplets or particles; possible heterogeneity in multiphase systems Limited improvement in hydrophobicity; moderate or inconsistent WVP reduction	Simple, low cost, easy scale-up	Insufficient dispersion for lipids/nanofillers; aggregation risk	[126]
High-Pressure Homogenization (HPH)	High shear, turbulence, cavitation	Fine emulsification, droplet size reduction, interfacial stabilization	Uniform microstructure; small and stable lipid droplets Significant increase in WCA; marked WVP reduction	Excellent dispersion; reproducible barrier improvement	High energy consumption; equipment cost	[115,125]
Ultrasonic Homogenization	Localized very high energy (acoustic cavitation)	Nanofiller dispersion, deagglomeration, emulsification	Improved nanoparticle distribution; increased diffusion tortuosity Moderate–high WVP reduction; indirect hydrophobicity enhancement	Highly effective for nanocomposites; lab-scale precision	Risk of polymer degradation; limited scalability	[127]

Water vapor permeability (WVP); water contact angle (WCA).

### 5.2. Thermal and Shear Treatment During Film Formation

Thermal and shear conditions during casting, extrusion, or thermocompression directly control starch gelatinization, plasticizer distribution, and additive dispersion. Adequate thermo-mechanical input disrupts native granule structure and promotes formation of a continuous TPS network, improving barrier uniformity. Conversely, excessive temperature or shear may cause molecular degradation, plasticizer volatilization, or phase separation, resulting in inferior mechanical and barrier properties [15,115].

### 5.3. Post-Drying Conditioning and Physical Aging of Films

Post-processing conditions determines whether hydrophobicity enhancements achieved during formulation and starch-based film formation are preserved or lost over time. Accordingly, conditioning protocols should be explicitly reported and standardized when evaluating structure–property relationships in starch-based films. Relative humidity (RH) and temperature during storage strongly influence moisture sorption, retrogradation (physical aging), plasticizer redistribution, and phase reorganization, all of which affect hydrophobicity and permeability of starch-based films. Conditioning under controlled or low relative humidity promotes matrix densification and stabilizes hydrophobic domains, improving reproducibility of WVP and mechanical properties. In contrast, high humidity accelerates water uptake, increases chain mobility, and degrades barrier performance [15,115].

Controlled-storage studies on films from different starch sources show that crystallinity, Tg, and WVP evolve significantly over weeks to months, and that the direction and magnitude of WVP change depend strongly on plasticizer level and storage conditions (RH, temperature) [128,129]. More recent aging studies further confirm that retrogradation at different temperatures produces measurable, time-dependent shifts in barrier and mechanical properties, illustrating that “best” performance immediately after processing may not be the “stable” performance relevant to packaging service conditions [129].

Plasticizer migration and redistribution is another major durability risk, particularly in glycerol-plasticized TPS. Glycerol and water act as coupled plasticizers: changes in ambient RH drive moisture sorption/desorption, which modifies local mobility and can create concentration gradients that promote plasticizer redistribution. This not only alters bulk permeability (via free volume and chain mobility), but can also change surface composition, shifting WCA over time. If glycerol enriches at the surface (or if water is sorbed at high RH), surface energy tends to increase and WCA decreases; conversely, plasticizer loss to the environment or inward redistribution can yield transient increases in WCA that do not necessarily correspond to improved bulk barrier performance [130].

Storage temperature fluctuations further intensify these effects by accelerating diffusion kinetics and shifting sorption equilibria. Temperature cycling can increase molecular mobility intermittently, amplifying retrogradation, enabling faster plasticizer redistribution, and destabilizing dispersed hydrophobic phases or interfacial architectures formed during processing. In practical packaging scenarios—where films experience variable temperatures (e.g., warehouse → transport → retail)—durability must therefore be evaluated under application-relevant thermal and humidity histories. The sensitivity of starch-based films to RH and temperature, including associated changes in permeability and mechanical behavior, has been repeatedly highlighted as a central barrier to reliable service performance [131].

Durability concerns are particularly acute for processing-enabled hydrophobicity strategies that rely on microstructural organization rather than permanent chemical fixation—such as surface-enriched lipids/waxes, emulsified oils, or percolated filler networks. During aging, dispersed hydrophobic domains can coarsen (Ostwald ripening/coalescence), bloom to the surface, or reorganize under humidity-driven plasticization, leading to drift in both WCA and WVP. Similarly, nanofiller-based tortuosity effects can weaken if interfacial regions restructure or if moisture disrupts filler–matrix interactions, especially in systems lacking strong compatibilization. These considerations imply that claims of “enhanced hydrophobicity” should be supported not only by initial WVP/WCA measurements, but also by retention metrics (e.g., ΔWCA and ΔWVP after defined storage periods, humidity conditioning, and temperature cycling), alongside structural diagnostics (DSC/XRD for retrogradation, gravimetry/DMA for mobility changes, and surface-sensitive analyses for compositional drift).

## 6. Scale-Up Feasibility, Industrial Compatibility, Cost, and Process Stability

While numerous strategies markedly improve the hydrophobicity of starch-based films at laboratory scale, their industrial relevance depends on compatibility with continuous processing, economic feasibility, long-term property stability, and compliance with food-contact regulations. In this context, scale-up feasibility is closely tied to the processing route.

Solution casting offers high formulation flexibility but is limited by solvent handling, drying energy demands, and batch variability, whereas melt-based technologies—such as extrusion, blown-film extrusion, thermocompression, and coextrusion—are inherently more compatible with industrial throughput and quality control requirements [61,63]. Among these, extrusion-based routes are particularly attractive because they enable continuous film production and in-line compounding of plasticizers, compatibilizers, and additives. Improved barrier and processing performance of extruded TPS films has been achieved through careful control of melt rheology and plasticizer chemistry [61]. Reactive extrusion further enables solvent-free chemical modification, including citric-acid-mediated crosslinking, offering an industrially viable route to network fixation and moisture resistance, if residence time, moisture content, and thermal history are tightly controlled to avoid degradation or discoloration [129,130].

Hydrophobic additives, including waxes, lipids, and oils, are among the most industrially accessible approaches because they preserve the native starch backbone and can be readily incorporated by compounding. However, their scalability is frequently limited by dispersion control and long-term morphological stability. Droplet coalescence, phase separation, and additive blooming can induce surface heterogeneity and time-dependent drift in WCA and WVP, particularly under humidity and temperature fluctuations. Consequently, scalable additive-based strategies typically require compatibilization or multilayer architectures that physically stabilize hydrophobic domains.

Reinforcement with nanofillers can substantially enhance barrier properties through increased diffusion tortuosity and matrix densification, yet industrial feasibility is constrained by supply-chain maturity, production cost, and energy intensity. This is particularly evident for nanocellulosic reinforcements, whose large-scale adoption is limited by high capital and energy requirements for fibrillation, viscosity constraints at high solids content, and the need for consistent quality across batches [131,132]. Accordingly, nanofiller strategies are most scalable when based on low-cost feedstocks, industrially viable dispersion methods, and demonstrable long-term property retention.

Multilayer and multiphase film architectures represent one of the most robust scale-up pathways, as they are compatible with coextrusion, lamination, and coating operations already established in the packaging industry. Multilayer TPS/PLA and TPS/PBAT systems fabricated through scalable processing routes have shown simultaneous improvements in moisture and oxygen barrier performance while maintaining mechanical integrity and transparency [133,134].

## 7. Practical Packaging Applications and Regulatory Considerations

Scalable starch-based packaging materials must balance durable hydrophobicity, controlled gas permeability, process compatibility, and regulatory compliance, rather than maximize any single performance metric. For moisture-sensitive dry foods (e.g., cereals, powders, bakery snacks), low WVP is required to prevent caking and texture loss. For oxygen- and aroma-sensitive products (e.g., nuts, coffee), oxygen transmission rate becomes the dominant design parameter, making nanofillers, nanoclays, and multilayer or blended architectures particularly relevant, provided dispersion stability and humidity resistance [135].

Packaging materials intended for food contact are regulated under Regulation (EC) No 1935/2004 [136], which requires food-grade components and limits harmful migration; while native starch is generally regarded as safe, plasticizers and chemical modifications must be carefully controlled, as low-molecular-weight additives may migrate and affect both food quality and long-term material performance.

## 8. Conclusions

This review highlights that the intrinsic hydrophilicity of starch-based films—rooted in the high density of hydroxyl groups and the supramolecular organization of amylose and amylopectin—poses significant limitations in terms of moisture sensitivity, barrier performance, and mechanical stability. Over the past decade, substantial progress has been made in addressing these limitations through chemical, physical, and structural modification strategies, including: (i) chemical modification (especially esterification/crosslinking), which most directly reduces hydrophilicity and improves WCA and barrier performance but must be carefully controlled to avoid losses in flexibility, processability, or food-contact compliance; (ii) hydrophobic additives (waxes, oils, lipids), which can raise surface hydrophobicity and lower WVP, although performance depends on compatibility and dispersion and may be undermined by phase separation; and (iii) fiber/nanocellulose reinforcement, which improves tortuosity and mechanical integrity and can support moisture-barrier gains, particularly when fibers are surface-modified.

Overall, no single strategy fully overcomes native starch limitations. The most effective systems reported from 2015–2025 are hybrid formulations combining chemical modification with hydrophobic phases and nanostructured reinforcements, leveraging complementary mechanisms to achieve better hydrophobicity, balanced mechanics, and retained biodegradability.

Future research should therefore prioritize the rational design of synergistic starch-based systems, with particular emphasis on interfacial chemistry, dispersion control, and the coupling of hydrophobicity enhancement with long-term mechanical and barrier stability. Additionally, scalable processing routes, life-cycle assessment, and performance evaluation under realistic service conditions will be essential to bridge the gap between laboratory-scale developments and industrial implementation. Through such integrated and application-oriented approaches, starch-based films can progress from promising biodegradable materials to viable alternatives for sustainable packaging and related applications.

## Figures and Tables

**Table 1:** Processing routes for starch-based films and their implications for hydrophobicity enhancement.

Processing Route	Typical Scale	Key Advantages	Main Limitations	Implications for Hydrophobicity
Solution casting	Laboratory	Simple; precise formulation control	Long drying time; high water use; limited scalability	Sensitive to drying-induced migration; good for screening hydrophobic additives
Extrusion/ film extrusion	Industrial	Continuous; scalable; good dispersion under shear	Narrow processing window; moisture control critical	Enables bulk barrier improvement if dispersion is optimized
Thermocompression	Pilot/ industrial	Dense films; controlled thickness	Requires pre-processed TPS	Often improves barrier via densification
Reactive extrusion	Emerging industrial	Solvent-free; integrated modification	High sensitivity to residence time and moisture	Strong potential for durable hydrophobicity via network fixation

**Table 2:** Main limitations of native starch-based films and their molecular and structural origins.

Limitation	Macroscopic Manifestation	Molecular/ Structural Origin	Key Consequences for Applications
High hydrophilicity	Low WCA (<60°); rapid moisture uptake	High density of hydroxyl (–OH) groups along amylose and amylopectin chains promotes strong starch–water hydrogen bonding	Poor resistance to humidity and liquid water; unsuitable for moist foods
High water solubility	Partial or complete dissolution in aqueous environments	Disruption of starch–starch hydrogen bonds by water molecules; amorphous regions highly accessible to diffusion	Loss of film integrity; limited use in aqueous or high-humidity conditions
High WVP	Inefficient moisture barrier	Hydrophilic polymer matrix with low tortuosity and high free volume	Poor shelf-life protection in food packaging
Brittleness	Low EB; brittle fracture	Strong intermolecular hydrogen bonding and lack of flexible chain segments	Mechanical failure during handling and processing
Low TS	Limited load-bearing capacity	Weak intermolecular cohesion under hydrated conditions; plasticization by absorbed water	Restricted use in structural or protective packaging
Dimensional instability	Swelling, shrinkage, and warping under humidity changes	Water-induced expansion of amorphous regions and disruption of crystalline domains	Poor shape retention during storage and transport
Retrogradation	Increased stiffness, opacity, and embrittlement over time	Recrystallization and reassociation of amylose and amylopectin chains	Deterioration of long-term mechanical and barrier properties
Plasticizer-induced moisture sensitivity	Increased moisture content and WVP after plasticization	Hygroscopic nature of glycerol, sorbitol, and PEG; enhanced water binding	Trade-off between flexibility and water resistance
Plasticizer migration	Surface stickiness; loss of mechanical properties over time	Phase separation and diffusion of low-molecular-weight plasticizers	Reduced durability and shelf stability

Water vapor permeability (WVP); water contact angle (WCA); tensile strength (TS); elongation at break (EB); polyethylene glycol (PEG).

**Table 3:** Modification strategies to improve hydrophobicity of starch-based films.

Modification Strategy	Targeted Limitation(s)	Mechanism of Improvement	Improvements
Chemical crosslinking (e.g., citric acid)	High hydrophilicity; high solubility; dimensional instability	Formation of covalent ester bonds reduces free hydroxyl groups and restricts chain mobility	↓ Water solubility ↓ WVP; ↑ WCA; improved dimensional stability
Esterification with long-chain reagents	Low water resistance; low surface hydrophobicity	Substitution of –OH groups with hydrophobic alkyl chains; surface energy reduction	WCA > 100° ↓ moisture uptake
Grafting of hydrophobic polymers	Poor barrier properties; moisture sensitivity	Introduction of hydrophobic side chains and microphase separation	↑ WCA; ↓ WVP
Incorporation of natural waxes (beeswax, candelilla)	High WVP; low WCA	Formation of hydrophobic domains and increased surface roughness	↑ WCA; ↓ WVP
Incorporation of vegetable or essential oils	High moisture uptake; poor barrier properties	Reduction in hydrophilic site accessibility; disruption of hydrogen bonding	↓ moisture content; ↓ WVP (at optimal oil content)
Nanoparticle reinforcement (ZnO, AgO)	High WVP; poor moisture barrier	Increased tortuosity and surface roughening; barrier path elongation	↓ WVP; ↑ WCA
Cellulose nanocrystals	Low mechanical strength; high permeability	High crystallinity and strong interfacial hydrogen bonding increase matrix tortuosity	↑ TS; ↓ WVP; moderate ↑ WCA
Cellulose nanofibrils	Brittleness; poor mechanical stability	Formation of entangled fibrillar network reinforcing matrix cohesion	↑ TS; ↓ moisture diffusion
Chemical modification of fibers (e.g., tannic acid, acid treatment)	Fiber-induced hydrophilicity; interfacial incompatibility	Reduction in surface polarity and improved fiber–matrix adhesion	↑ WCA; ↓ moisture absorption
Hybrid strategies (e.g., wax + nanocellulose)	Multiple limitations simultaneously	Synergistic effects combining hydrophobic domains and tortuous diffusion pathways	↑ WCA; ↓ WVP; balanced mechanical properties

Water vapor permeability (WVP); water contact angle (WCA); tensile strength (TS).

**Table 4:** Typical ranges of hydrophobicity and barrier performance reported for chemically modified starch-based films.

Strategy	WCA ^1^	WVP (g m^−1^ s^−1^ Pa^−1^) ^1^	Impact	Representative Literature Review
Native TPS	20–60°	1.2 × 10^−7^ — 8.3 × 10^−5^	Highly hydrophilic; poor moisture barrier	[57,75,76]
Citric acid (CA) crosslinking	50–80°	(1–4) × 10^−10^	Reduced solubility and WVP at optimal CA content; excessive CA may plasticize	[22,77,78,79]
Esterification (short-chain, e.g., acetylation)	60–90°	(0.8–3) × 10^−10^	Moderate hydrophobicity improvement; depends on degree of substitution	[80,81,82]
Esterification (long-chain hydrophobic moieties)	90–120°	(0.5–2) × 10^−10^	Strong hydrophobicity; increased surface roughness and polarity reduction	[82,83]
Combined chemical modification + mild plasticization	70–100°	(0.7–3) × 10^−10^	Balanced hydrophobicity and flexibility	[84]

Water vapor permeability (WVP); water contact angle (WCA). ^1^ The reported variability arises from differences in test conditions and composition across the referenced studies.

**Table 5:** Comparative overview of hydrophobic additives used to improve the moisture resistance of starch-based films.

Hydrophobic Additive Class	WCA ^1^	WVP Reduction (Mean Values) ^1^	Dominant Mechanisms	Key Advantages	Main Limitations	Representative Literature Review
Natural waxes (beeswax, candelilla, carnauba)	80–110	20–60% (0.8–3.5) × 10^−10^ g·m^−1^·s^−1^·Pa^−1^	Surface-energy reduction; surface roughness; hydrophobic domain formation	Food-grade; strong surface hydrophobicity; scalable	Phase separation; brittleness at high loading; dispersion challenges	[87,88]
Vegetable oils (soybean, sunflower, rice bran)	60–95	15–45% (1.5–4.0) × 10^−10^ g·m^−1^·s^−1^·Pa^−1^	Hydroxyl shielding; partial plasticization; hydrophobic phase dispersion	Low cost; improves flexibility; food compatibility	Migration during storage; limited long-term barrier stability	[89]
Essential oils (clove, oregano, geranium)	65–95	20–50% (1.2–3.8) × 10^−10^ g·m^−1^·s^−1^·Pa^−1^	Hydrophobic domains + antimicrobial activity	Active packaging functionality; moderate hydrophobicity	Volatility; aroma transfer; stability issues	[89]
Pickering-stabilized lipid systems	90–120	40–70%	Stable hydrophobic domains; controlled spatial distribution	Superior dispersion; improved stability	More complex formulation	[87,88]
Hydrophobic nanoparticles (ZnO, SiO_2_, organoclays)	80–120	30–70% (0.7–2.5) × 10^−10^ g·m^−1^·s^−1^·Pa^−1^	Diffusion-path tortuosity; surface roughening	Strong bulk barrier improvement; multifunctionality	Agglomeration risk; opacity; regulatory concerns	[90]
Carbon-based nanomaterials (CNFs, CNCs, MMT, O-MMT)	85–120	40–75% (0.6–2.0) × 10^−10^ g·m^−1^·s^−1^·Pa^−1^	High-aspect-ratio tortuosity; interfacial reinforcement	Excellent WVP reduction at low loadings	Cost; dispersion difficulty; sustainability concerns	[91]
Multilayer/gradient architectures	90–120	35–65%	Surface-localized hydrophobicity	High WCA with low additive content	Additional processing steps	[92]

Cellulose Nanofibers (CNFs); Cellulose Nanocrystals (CNCs); Natural Montmorillonite (MMT); Organically Modified Montmorillonite (O-MMT). ^1^ The reported variability arises from differences in test conditions and composition across the referenced studies.

**Table 6:** Typical effects of cellulose nanocrystals (CNCs) and cellulose nanofibrils (CNFs) on hydrophobicity and mechanical properties of starch-based films.

Nanocellulose	WCA ^1^	WVP ^1^ (g·m^−1^·s^−1^·Pa^−1^)	TS ^1^ (MPa)	Mechanisms	Representative Literature Review
CNCs	65–95°	(0.8–3.0) × 10^−10^	5–35	↑ tortuosity; high crystallinity restricts diffusion pathways	[26]
CNFs	60–90°	(1.0–3.5) × 10^−10^	10–45	Entangled fibrillar network; strong interfacial bonding and stress transfer	[26,115]

Water vapor permeability (WVP); water contact angle (WCA); tensile strength (TS). ^1^ The reported variability arises from differences in test conditions and composition across the referenced studies.

## Data Availability

The original contributions presented in this study are included in the article. Further inquiries can be directed to the corresponding author.

## Abbreviations

The following abbreviations are used in this manuscript:

TPS	Thermoplastic starch
TS	Tensile strength
YM	Young’s modulus
EAB	Elongation at Break
WVP	Water vapor permeability
WCA	Water contact angle

## References

[1] Gautam B.P.S., Qureshi A., Gwasikoti A., Kumar V., Gondwal M., Soni R., Debbarma P., Suyal D.C., Goel R. (2024). Global scenario of plastic production, consumption, and waste generation and their impacts on environment and human health. Advanced Strategies for Biodegradation of Plastic Polymers.

[2] Anwar M., jacnova M.E., Dastgir S. (2025). Circular plastic economy for sustainable development: Current advances and future perspectives. RSC Sustain..

[3] Watt E., Picard M., Maldonado B., Abdelwahab M.A., Mielewski D.F., Drzal L.T., Mohanty A.K. (2021). Ocean plastics: Environmental implications and potential routes for mitigation—A perspective. RSC Adv..

[4] Pottinger A.S., Geyer R., Biyani N., Martinez C.C., Nathan N., Morse M.R., McCauley D.J. (2024). Pathways to reduce global plastic waste mismanagement and greenhouse gas emissions by 2050. Science.

[5] Mishra A.K., Singh J., Mishra P.P. (2021). Microplastics in polar regions: An early warning to the world’s pristine ecosystem. Sci. Total Environ..

[6] Das T., Das N., Zuthi M.F.R., Pal S.K., Kraft E., Haupt T., Kuehlewindt S. (2025). Plastic waste in marine ecosystems: Identification techniques and policy interventions. Water Air Soil Pollut..

[7] Visco A., Scolaro C., Facchin M., Brahimi S., Belhamdi H., Gatto V., Beghetto V. (2022). Agri-food wastes for bioplastics: European prospective on possible applications in their second life for a circular economy. Polymers.

[8] Dilshad E., Waheed H., Ali U., Amin A., Ahmed I., Kuddus M., Roohi (2021). General structure and classification of bioplastics and biodegradable plastics. Bioplastics for Sustainable Development.

[9] Arruda T.R., Machado G.D.O., Marques C.S., Souza A.L.D., Pelissari F.M., Oliveira T.V.D., Silva R.R.A. (2025). An overview of starch-based materials for sustainable food packaging: Recent advances, limitations, and perspectives. Macromol.

[10] Vicente D., Proença D.N., Morais P.V. (2023). The role of bacterial polyhydroalkanoate (PHA) in a sustainable future: A review on the biological diversity. Int. J. Environ. Res. Public Health.

[11] Santhosh R., Ahmed J., Thakur R., Sarkar P. (2024). Starch-based edible packaging: Rheological, thermal, mechanical, microstructural, and barrier properties—A review. Sustain. Food Technol..

[12] Zhu F. (2015). Impact of ultrasound on structure, physicochemical properties, modifications, and applications of starch. Trends Food Sci. Technol..

[13] Zhu W., Zhang D., Liu X., Ma T., He J., Dong Q., Cai J. (2022). Improving the hydrophobicity and mechanical properties of starch nanofibrous films by electrospinning and cross-linking for food packaging applications. LWT.

[14] Koch K. (2018). Starch-based films. Starch in Food.

[15] Agarwal S. (2021). Major factors affecting the characteristics of starch based biopolymer films. Eur. Polym. J..

[16] Dorantes-Fuertes M.G., López-Méndez M.C., Martínez-Castellanos G., Meléndez-Armenta R.Á., Jiménez-Martínez H.E. (2024). Starch extraction methods in tubers and roots: A systematic review. Agronomy.

[17] Ribba L., Garcia N.L., D’Accorso N., Goyanes S. (2017). Disadvantages of starch-based materials, feasible alternatives in order to overcome these limitations. Starch-Based Materials in Food Packaging: Processing, Characterization and Applications.

[18] Muñoz-Gimena P.F., Oliver-Cuenca V., Peponi L., López D. (2023). A review on reinforcements and additives in starch-based composites for food packaging. Polymers.

[19] Kalita P., Bora N.S., Gogoi B., Goswami A., Pachuau L., Das P.J., Roy S. (2025). Improving the hydrophobic nature of biopolymer based edible packaging film: A review. Food Chem..

[20] Nazrin A., Ilyas R.A., Rajeshkumar L., Hazrati K.Z., Jamal T., Mahardika M., Radzi A.M. (2025). Lignocellulosic fiber-reinforced starch thermoplastic composites for food packaging application: A review. Food Packag. Shelf Life.

[21] Fatima S., Khan M.R., Ahmad I., Sadiq M.B. (2024). Recent advances in modified starch based biodegradable food packaging: A review. Heliyon.

[22] Kalu A.O., Omonije O.O., Egwim E.C., Jigam A.A., Muhammad H.L. (2025). Crosslinking modification for starch and starch-based films (a review). Starch/Stärke.

[23] Xie Q., Fan B., Tao R., Wang F., Sun Y. (2025). A comprehensive review of starch: Structure, properties, chemical modification, and application in food preservation. Food Front..

[24] Gao S., Liu R., Song H., Huang D., Dai Y., Hou H., Wang W. (2024). Regulation of fine structure of different types of natural waxes by octenyl succinate starch in starch bioplastics. Food Biosci..

[25] Rammak T., Boonsuk P., Kaewtatip K. (2021). Mechanical and barrier properties of starch blend films enhanced with kaolin for application in food packaging. Int. J. Biol. Macromol..

[26] Singh G.P., Bangar S.P., Yang T., Trif M., Kumar V., Kumar D. (2022). Effect on the properties of edible starch-based films by the incorporation of additives: A review. Polymers.

[27] Shapi’i R.A., Othman S.H., Basha R.K., Naim M.N. (2022). Mechanical, thermal, and barrier properties of starch films incorporated with chitosan nanoparticles. Nanotechnol. Rev..

[28] Mukaila T., Adeniyi A., Bello I., Sarker N.C., Monono E., Hammed A. (2024). Optimizing film mechanical and water contact angle properties via PLA/starch/lecithin concentrations. Clean. Circ. Bioeconomy.

[29] Żołek-Tryznowska Z., Jeznach A., Bednarczyk E., Murawski T., Piłczyńska K., Sikora S., Tryznowski M. (2024). Effect of hydrophobic nano-silica content on the surface properties of corn-starch films. Ind. Crops Prod..

[30] Abdullah, Cai J., Hafeez M.A., Wang Q., Farooq S., Huang Q., Xiao J. (2022). Biopolymer-based functional films for packaging applications: A review. Front. Nutr..

[31] Jayarathna S., Andersson M., Andersson R. (2022). Recent advances in starch-based blends and composites for bioplastics applications. Polymers.

[32] Cui C., Gao L., Dai L., Ji N., Qin Y., Shi R., Sun Q. (2023). Hydrophobic biopolymer-based films: Strategies, properties, and food applications. Food Eng. Rev..

[33] Trinh B.M., Chang C.C., Mekonnen T.H. (2021). Facile fabrication of thermoplastic starch/poly (lactic acid) multilayer films with superior gas and moisture barrier properties. Polymer.

[34] Bayer I.S. (2021). Biopolymers in multilayer films for long-lasting protective food packaging: A review. Sustainable Food Packaging Technology.

[35] Kiattijiranon P., Auras R.A., Sane A. (2024). Enhanced functional properties for packaging applications using sodium alginate/starch bilayer and multilayer films. ACS Appl. Polym. Mater..

[36] Amaral L., Rodrigues F., Silva A., Costa P., Delerue-Matos C., Vieira E.F. (2022). Reinforcement of starch film with *Castanea sativa* shells polysaccharides: Optimized formulation and characterization. Food Chem..

[37] Freitas G.P., Cunha P.I., Maia A.A., Santos D.S., Lorevice M.V., Gouveia R.F. (2024). Starch-based films: Tuning physical properties driven by nanocellulose–natural rubber latex composites. Ind. Crops Prod..

[38] Siddiqui S.A., Yang X., Deshmukh R.K., Gaikwad K.K., Bahmid N.A., Munoz R.C. (2024). Recent advances in reinforced bioplastics for food packaging—A critical review. Int. J. Biol. Macromol..

[39] Silva S.B., Freitas O.M., Vieira E.F., Delerue-Matos C., Domingues V.F. (2025). Valorization of chestnut processing wastes into bio-based composites and bioplastics: A review. Polym. Compos..

[40] Tan S.X., Andriyana A., Ong H.C., Lim S., Pang Y.L., Ngoh G.C. (2022). Roles of nanofillers and plasticizers towards sustainable starch-based bioplastic fabrication. Polymers.

[41] Suleman A., Bora J., Kalita A.J., Chetia B., Visakh P.M. (2025). Starch-based nanocomposite for food packaging applications. Engineering Applications of Polymer-Based Nanocomposites.

[42] Basavegowda N., Baek K.H. (2021). Advances in functional biopolymer-based nanocomposites for active food packaging applications. Polymers.

[43] Hakke V.S., Landge V.K., Sonawane S.H., Babu G.U.B., Ashokkumar M., Flores E.M. (2022). Physical, mechanical, thermal and barrier properties of starch nanoparticle/polyurethane nanocomposite films synthesized by an ultrasound-assisted process. Ultrason. Sonochem..

[44] Kesari A.K., Mandava S., Munagala C.K., Nagar H., Aniya V. (2022). DES-ultrasonication processing for cellulose nanofiber and its compounding in biodegradable starch-based packaging films through extrusion. Ind. Crops Prod..

[45] Huang X., Chen L., Liu Y. (2024). Effects of ultrasonic and ozone modification on the morphology, mechanical, thermal and barrier properties of corn starch films. Food Hydrocoll..

[46] Mileti O., Mammolenti D., Baldino N., Lupi F.R., Gabriele D. (2024). Starch films loaded with tannin: Study of rheological and physical properties. Int. J. Biol. Macromol..

[47] Thakur R., Pristijono P., Scarlett C.J., Bowyer M., Singh S.P., Vuong Q.V. (2019). Starch-based films: Major factors affecting their properties. Int. J. Biol. Macromol..

[48] Bertoft E. (2017). Understanding starch structure: Recent progress. Agronomy.

[49] Zou Y., Yuan C., Cui B., Liu P., Wu Z., Zhao H. (2021). Formation of high amylose corn starch/konjac glucomannan composite film with improved mechanical and barrier properties. Carbohydr. Polym..

[50] Zhong Y., Tai L., Blennow A., Ding L., Herburger K., Qu J., Liu X. (2023). High-amylose starch: Structure, functionality and applications. Crit. Rev. Food Sci. Nutr..

[51] Hsieh C.F., Liu W., Whaley J.K., Shi Y.C. (2019). Structure and functional properties of waxy starches. Food Hydrocoll..

[52] Sengupta A., Chakraborty I., Mazumder N. (2023). An insight into the physicochemical characterisation of starch-lipid complex and its importance in food industry. Food Rev. Int..

[53] Domene-López D., García-Quesada J.C., Martin-Gullon I., Montalbán M.G. (2019). Influence of starch composition and molecular weight on physicochemical properties of biodegradable films. Polymers.

[54] Wang Y., Ju J., Diao Y., Zhao F., Yang Q. (2025). The application of starch-based edible film in food preservation: A comprehensive review. Crit. Rev. Food Sci. Nutr..

[55] Jia H., Ren F., Liu H. (2025). Innovative non-thermal processing: Unraveling structural and functional transformations in food macromolecules—Starch, proteins, and lipids. Food Res. Int..

[56] Yu W., Yu Y., Li J., Liang H., Li Y., Li B. (2025). Effects of deacetylated konjac glucomannan on retrogradation properties of pea, mung bean and potato starches during storage. Int. J. Biol. Macromol..

[57] Müller C.M.O., Laurindo J.B., Yamashita F. (2009). Effect of cellulose fibers addition on the mechanical properties and water vapor barrier of starch-based films. Food Hydrocoll..

[58] Chang Q., Zheng B., Zhang Y., Zeng H. (2021). A comprehensive review of the factors influencing the formation of retrograded starch. Int. J. Biol. Macromol..

[59] Żołek-Tryznowska Z., Kałuża A. (2021). The influence of starch origin on the properties of starch films: Packaging performance. Materials.

[60] Bangar S.P., Purewal S.S., Trif M., Maqsood S., Kumar M., Manjunatha V., Rusu A.V. (2021). Functionality and applicability of starch-based films: An eco-friendly approach. Foods.

[61] Dang K.M., Yoksan R. (2021). Thermoplastic starch blown films with improved mechanical and barrier properties. Int. J. Biol. Macromol..

[62] Qiu C., Hu H., Chen B., Lin Q., Ji H., Jin Z. (2024). Research progress on the physicochemical properties of starch-based foods by extrusion processing. Foods.

[63] Tavassoli M., Bahramian B., Majlesi M., Ghaderi S., Roy S., Shahid M. (2026). Starch-Based polymers in food packaging: Recent advances. Biomaterials for Sustainable Food Packaging. Environmental Footprints and Eco-Design of Products and Processes.

[64] Shakil F. (2024). Reactive extrusion: Filled polymer compounds and its applications. Polymer Composites: From Computational to Experimental Aspects.

[65] González-Torres B., Robles-García M.Á., Gutiérrez-Lomelí M., Padilla-Frausto J.J., Navarro-Villarruel C.L., Del-Toro-Sánchez C.L., Reynoso-Marín F.J. (2021). Combination of sorbitol and glycerol, as plasticizers, and oxidized starch improves physicochemical characteristics of films for food preservation. Polymers.

[66] Abera G., Woldeyes B., Demash H.D., Miyake G. (2020). The effect of plasticizers on thermoplastic starch films developed from Anchote starch. Int. J. Biol. Macromol..

[67] Ren J., Zhang W., Lou F., Wang Y., Guo W. (2016). Characteristics of starch-based films produced using glycerol and 1-butyl-3-methylimidazolium chloride as combined plasticizers. Starch/Stärke.

[68] Domene-López D., Delgado-Marín J.J., García-Quesada J.C., Martín-Gullón I., Montalbán M.G. (2020). Electroconductive starch/multiwalled carbon nanotube films plasticized by 1-ethyl-3-methylimidazolium acetate. Carbohydr. Polym..

[69] Saberi B., Thakur R., Vuong Q.V., Chockchaisawasdee S., Golding J.B., Scarlett C.J., Stathopoulos C.E. (2016). Optimization of physical and optical properties of biodegradable edible films based on pea starch and guar gum. Ind. Crops Prod..

[70] Podshivalov A., Zakharova M., Glazacheva E., Uspenskaya M. (2017). Gelatin/potato starch edible biocomposite films: Correlation between morphology and physical properties. Carbohydr. Polym..

[71] Ortega-Toro R., Collazo-Bigliardi S., Roselló J., Santamarina P., Chiralt A. (2017). Antifungal starch-based edible films containing Aloe vera. Food Hydrocoll..

[72] Cano A., Chafer M., Chiralt A., González-Martínez C., Thakur V.K., Thakur M.K., Kessler M.R. (2017). Strategies to improve the functionality of starch-based films. Handbook of Composites from Renewable Materials, Vol. 4: Functionalization.

[73] Sethi S., Choudhary P., Nath P., Chauhan O.P., Chauhan O.P. (2022). Starch gelatinization and modification. Advances in Food Chemistry.

[74] (2002). Standard Test Method for Tensile Properties of Thin Plastic Sheeting. Annual Book of Astm Standards.

[75] Gutiérrez T.J., Ollier R., Alvarez V.A. (2017). Surface properties of thermoplastic starch materials reinforced with natural fillers. Functional Biopolymers.

[76] Zhang M., Jia H., Wang B., Ma C., He F., Fan Q., Liu W. (2023). A prospective review on the research progress of citric acid modified starch. Foods.

[77] Hsu Y.-I. (2025). Development of functional degradable materials by precise crosslinking design of biobased polymers. Polym. J..

[78] Kumar Y., Singh S., Saxena D.C. (2025). A comprehensive review on methods, mechanisms, properties, and emerging applications of crosslinked starches. Int. J. Biol. Macromol..

[79] Ruhul Amin M., Anannya F.R., Mahmud M.A., Raian S. (2020). Esterification of starch in search of a biodegradable thermoplastic material. J. Polym. Res..

[80] Xu J., Andrews T.D., Shi Y.C. (2020). Recent advances in the preparation and characterization of intermediately to highly esterified and etherified starches: A review. Starch/Stärke.

[81] Li X., Gao J., Chen W., Liang J., Gao W., Bodjrenou D.M., Zheng B. (2025). Properties and functions of acylated starch with short-chain fatty acids: A comprehensive review. Crit. Rev. Food Sci. Nutr..

[82] Sinhmar A., Pathera A.K., Sharma S., Nehra M., Thory R., Nain V. (2023). Impact of various modification methods on physicochemical and functional properties of starch: A review. Starch/Stärke.

[83] Matheus J.R.V., Dalsasso R.R., Rebelatto E.A., Andrade K.S., Andrade L.M.D., Andrade C.J.D., Fai A.E.C. (2023). Biopolymers as green-based food packaging materials: A focus on modified and unmodified starch-based films. Compr. Rev. Food Sci. Food Saf..

[84] Karma V., Gupta A.D., Yadav D.K., Singh A.A., Verma M., Singh H. (2022). Recent developments in starch modification by organic acids: Aeview. Starch/Stärke.

[85] Zahid M.K., Ahmad D., Amin R., Bao J. (2025). Sorghum starch: Composition, structure, functionality, and strategies for its improvement. Compr. Rev. Food Sci. Food Saf..

[86] Galus S., Kadzińska J. (2015). Food applications of emulsion-based edible films and coatings. Trends Food Sci. Technol..

[87] Cheng Y., Cai X., Zhang X., Zhao Y., Song R., Xu Y., Gao H. (2024). Applications in Pickering emulsions of enhancing preservation properties: Current trends and future prospects in active food packaging coatings and films. Trends Food Sci. Technol..

[88] Zhu F. (2019). Starch based Pickering emulsions: Fabrication, properties, and applications. Trends Food Sci. Technol..

[89] Gupta R.K., Pipliya S., Patel J., Srivastav P.P., Castro-Muñoz R., Wang C.K., Nemţanu M.R. (2025). Essential oil-embedded starch based films for active packaging: Insights into migration, stability, and preservation efficiency. Trends Food Sci. Technol..

[90] Akhila V., Badwaik L.S. (2022). Recent advancement in improvement of properties of polysaccharides and proteins based packaging film with added nanoparticles: A review. Int. J. Biol. Macromol..

[91] Rivadeneira-Velasco K.E., Utreras-Silva C.A., Díaz-Barrios A., Sommer-Márquez A.E., Tafur J.P., Michell R.M. (2021). Green nanocomposites based on thermoplastic starch: A review. Polymers.

[92] La Fuente Arias C.I., Kubo M.T.K.N., Tadini C.C., Augusto P.E.D. (2023). Bio-based multilayer films: A review of the principal methods of production and challenges. Crit. Rev. Food Sci. Nutr..

[93] Periyasamy T., Asrafali S.P., Lee J. (2025). Recent advances in functional biopolymer films with antimicrobial and antioxidant properties for enhanced food packaging. Polymers.

[94] Sun Q. (2018). Starch nanoparticles. Starch in Food.

[95] Minakawa A.F.K., Faria-Tischer P.C.S., Mali S. (2019). Simple ultrasound method to obtain starch micro- and nanoparticles from cassava, corn and yam starches. Food Chem..

[96] Patel C.M., Chakraborty M., Murthy Z.V.P. (2016). Fast and scalable preparation of starch nanoparticles by stirred media milling. Adv. Powder Technol..

[97] Apostolidis E., Mandala I. (2020). Modification of resistant starch nanoparticles using high-pressure homogenization treatment. Food Hydrocoll..

[98] Shang Y., Chao C., Yu J., Copeland L., Wang S., Wang S. (2018). Starch spherulites prepared by a combination of enzymatic and acid hydrolysis of normal corn starch. J. Agric. Food Chem..

[99] Dai L., Li C., Zhang J., Cheng F. (2018). Preparation and characterization of starch nanocrystals combining ball milling with acid hydrolysis. Carbohydr. Polym..

[100] Maryam, Kasim A., Novelina, Emriadi (2020). Preparation and characterization of sago (*Metroxylon* sp.) starch nanoparticles using hydrolysis-precipitation method. J. Phys. Conf. Ser..

[101] Harsanto B.W., Pranoto Y., Supriyanto, Kartini I. (2021). Breadfruit-based starch nanoparticles prepared using nanoprecipitation to stabilize a Pickering emulsion. J. Southwest Jiaotong Univ..

[102] Bernardo C.N., Kling I.C.S., Ferreira W.H., Andrade C.T., Simão R.A. (2022). Starch films containing starch nanoparticles as produced in a single step green route. Ind. Crops Prod..

[103] Oliveira A.V., da Silva A.P.M., Barros M.O., de Sá M., Souza Filho M., Rosa M.F., Azeredo H.M.C. (2018). Nanocomposite films from mango kernel or corn starch with starch nanocrystals. Starch/Stärke.

[104] Garavand Y., Taheri-Garavand A., Garavand F., Shahbazi F., Khodaei D., Cacciotti I. (2022). Starch-polyvinyl alcohol-based films reinforced with chitosan nanoparticles: Physical, mechanical, structural, thermal and antimicrobial properties. Appl. Sci..

[105] Amaregouda Y., Kamanna K. (2024). Carboxymethyl cellulose/starch-based films incorporating chitosan nanoparticles for multifunctional food packaging. Cellulose.

[106] Liu Q., Gao L., Qin Y., Ji N., Dai L., Xiong L., Sun Q. (2023). Incorporation of oxidized debranched starch/chitosan nanoparticles for enhanced hydrophobicity of corn starch films. Food Packag. Shelf Life.

[107] Zare Y. (2016). The roles of nanoparticles accumulation and interphase properties in properties of polymer particulate nanocomposites by a multi-step methodology. Compos. Part A Appl. Sci. Manuf..

[108] Anugrahwidya R., Armynah B., Tahir D. (2022). Composites bioplastic film for various concentration of zinc oxide (ZnO) nanocrystals towards physical properties for high biodegradability in soil and seawater. J. Polym. Environ..

[109] Babapour H., Jalali H., Mohammadi Nafchi A. (2021). The synergistic effects of zinc oxide nanoparticles and fennel essential oil on physicochemical, mechanical, and antibacterial properties of potato starch films. Food Sci. Nutr..

[110] Arezoo E., Mohammadreza E., Maryam M., Abdorreza M.N. (2020). The synergistic effects of cinnamon essential oil and nano TiO2 on antimicrobial and functional properties of sago starch films. Int. J. Biol. Macromol..

[111] Sani I.K., Geshlaghi S.P., Pirsa S., Asdagh A. (2021). Composite film based on potato starch/apple peel pectin/ZrO_2_ nanoparticles/microencapsulated *Zataria multiflora* essential oil: Investigation of physicochemical properties and use in quail meat packaging. Food Hydrocoll..

[112] Sun J., Wang S., Duan J.A., Xiao P. (2025). Eco-friendly processing and application of food-derived polysaccharides: Advances in extraction, separation and drying. Food Rev. Int..

[113] Nath D., Santhosh R., Pal K., Sarkar P. (2022). Nanoclay-based active food packaging systems: A review. Food Packag. Shelf Life.

[114] Ren J., Dang K., Pollet E., Avérous L. (2018). Preparation and characterization of thermoplastic potato starch/halloysite nano-biocomposites: Effect of plasticizer nature and nanoclay content. Polymers.

[115] Sun J., Yang X., Bai Y., Fang Z., Zhang S., Wang X., Guo Y. (2024). Recent advances in cellulose nanofiber modification and characterization and cellulose nanofiber-based films for eco-friendly active food packaging. Foods.

[116] Jonoobi M., Oladi R., Davoudpour Y., Oksman K., Dufresne A., Hamzeh Y., Davoodi R. (2015). Different preparation methods and properties of nanostructured cellulose from various natural resources and residues: A review. Cellulose.

[117] de Souza Coelho C.C., Silva R.B.S., Carvalho C.W.P., Rossi A.L., Teixeira J.A., Freitas-Silva O., Cabral L.M.C. (2020). Cellulose nanocrystals from grape pomace and their use for the development of starch-based nanocomposite films. Int. J. Biol. Macromol..

[118] Balakrishnan P., Gopi S., Thomas S. (2018). UV resistant transparent bionanocomposite films based on potato starch/cellulose for sustainable packaging. Starch/Stärke.

[119] Fazeli M., Keley M., Biazar E. (2018). Preparation and characterization of starch-based composite films reinforced by cellulose nanofibers. Int. J. Biol. Macromol..

[120] Chang C.C., Trinh B.M., Mekonnen T.H. (2021). Robust multiphase and multilayer starch/polymer (TPS/PBAT) film with simultaneous oxygen/moisture barrier properties. J. Colloid Interface Sci..

[121] Nair M., Tomar S., Punia W., Kukula-Koch W., Kumar M. (2020). Enhancing the functionality of chitosan- and alginate-based active edible coatings/films for the preservation of fruits and vegetables: A review. Int. J. Biol. Macromol..

[122] Kumar S., Mukherjee A., Dutta J. (2020). Chitosan based nanocomposite films and coatings: Emerging antimicrobial food packaging alternatives. Trends Food Sci. Technol..

[123] Ren L., Yan X., Zhou J., Tong J., Su X. (2017). Influence of chitosan concentration on mechanical and barrier properties of corn starch/chitosan films. Int. J. Biol. Macromol..

[124] Fonseca-García A., Jiménez-Regalado E.J., Aguirre-Loredo R.Y. (2021). Preparation of a novel biodegradable packaging film based on corn starch-chitosan and poloxamers. Carbohydr. Polym..

[125] Beikzadeh S., Ghorbani M., Shahbazi N., Izadi F., Pilevar Z., Mortazavian A.M. (2020). The effects of novel thermal and nonthermal technologies on the properties of edible food packaging. Food Eng. Rev..

[126] de Carvalho-Guimarães F.B., Correa K.L., de Souza T.P., Rodriguez Amado J.R., Ribeiro-Costa R.M., Silva-Júnior J.O.C. (2022). A review of Pickering emulsions: Perspectives and applications. Pharmaceuticals.

[127] Mi T., Zhang X., Liu P., Gao W., Li J., Xu N., Cui B. (2023). Ultrasonication effects on physicochemical properties of biopolymer-based films: A comprehensive review. Crit. Rev. Food Sci. Nutr..

[128] Xu W., Yan S., Xu X., Wang B., Abd El-Aty A.M. (2024). Investigation of film physical properties under various starch thermal treatments with emphasis on retrogradation effects. Food Chem..

[129] Basiak E., Lenart A., Debeaufort F. (2018). How glycerol and water contents affect the structural and functional properties of starch-based edible films. Polymers.

[130] Hazrati K.Z., Sapuan S.M., Zuhri M.Y.M., Jumaidin R. (2021). Effect of plasticizers on physical, thermal, and tensile properties of thermoplastic films based on *Dioscorea hispida* starch. Int. J. Biol. Macromol..

[131] Qin Y., Wang W., Zhang H., Dai Y., Hou H., Dong H. (2019). Effects of Citric Acid on Structures and Properties of Thermoplastic Hydroxypropyl Amylomaize Starch Films. Materials.

[132] Castro J.M., Montalbán M.G., Martínez-Pérez N., Domene-López D., Pérez J.M., Arrabal-Campos F.M., Fernández I., Martín-Gullón I., García-Quesada J.C. (2023). Thermoplastic starch/polyvinyl alcohol blends modification by citric acid–glycerol polyesters. Int. J. Biol. Macromol..

[133] Djafari Petroudy S.R., Chabot B., Loranger E., Naebe M., Shojaeiarani J., Gharehkhani S., Ahvazi B., Hu J., Thomas S. (2021). Recent Advances in Cellulose Nanofibers Preparation through Energy-Efficient Approaches: A Review. Energies.

[134] Signori-Iamin G., Aguado R.J., Putaux J.L., Santos A.F., Thielemans W., Delgado-Aguilar M. (2025). Energy and property trade-offs in nanocellulose production: High-pressure homogenization at different processing consistencies. Chem. Eng. J..

[135] Dong M., Mastroianni G., Bilotti E., Zhang H., Papageorgiou D.G. (2024). Biodegradable starch-based nanocomposite films with exceptional water and oxygen barrier properties. ACS Sustain. Chem. Eng..

[136] (2004). Regulation (EC) No 1935/2004 of the European Parliament and of the Council of 27 October 2004 on materials and articles intended to come into contact with food. Off. J. Eur. Union.

